# Lectures on Venereal and Other Diseases Arising from Sexual Intercousse

**Published:** 1850-09

**Authors:** 


### Part 2—Reviews and Notices of New Works.

### ARTICLE I.

*Lectures on Venereal and other Diseases arising from Sexual Intercourse.* Delivered in the summer of 1847, at the Hospital du Midi, Paris, by M. Ricord. Reported and translated by Victor de Meiric, M. D., M. R. C. S. S. Philadelphia: Ed, Barrington and Geo. D. Haswell, 1849; pp., 298; 12mo. (From the Publishers.) For sale by J. Keen, Jr., & Bro.

These lectures are already familiar to the readers of the *London Lancet*, having been published in that journal more than a year since. They have now been put into the form of a book which, being cheap, is accessible to the whole profession. There is no class of diseases concerning which practitioners generally have such crude and indefinite notions, as of Venereal Affections. In villages, Syphilis is rare, many practioners having been in active practice for years without seeing a half-dozen cases: nevertheless, it is the duty of the medical man to be prepared to manage the occasional case; and to that end, he cannot read a better book than the one before us. To Ricord is pre-eminently due the credit of having elucidated the nature and treatment of Venereal Diseases; and his sound views, based on the most extensive practice in the great Venereal Hospital of Paris, have become the guide in practice for most enlightened physicians and surgeons.

The present work contains his doctrines touching the nature and treatment of these affections, brought down to a very recent date, and any practitioner who desires to have the best treatise extant, on this subject, should not fail to procure and study this excellent little volume. M.

